# A Noninvasive Assessment of Tumor Proliferation in Lung cancer Patients using Intravoxel Incoherent Motion Magnetic Resonance Imaging

**DOI:** 10.7150/jca.48589

**Published:** 2021-01-01

**Authors:** Yu Zheng, Wenjun Huang, Xuelin Zhang, Chen Lu, Caixia Fu, Shihong Li, Guangwu Lin

**Affiliations:** 1 Department of Radiology, Huadong Hospital Affiliated to Fudan University, Shanghai, 200040, China.; 2 Department of Thoracic Surgery, Huadong Hospital Affiliated to Fudan University, Shanghai, 200040, China.; 3 Department of Pathology, Huadong Hospital Affiliated to Fudan University, Shanghai, 200040, China.; 4 Siemens Shenzhen Magnetic Resonance Ltd., Shenzhen, Guangdong Province, 518057, China.

**Keywords:** IVIM, Ki-67, MRI, NSCLC, SCLC, tumor differentiation

## Abstract

Ki-67 is a nuclear antigen widely used in routine pathologic analyses as a tumor cell proliferation marker for lung cancer. However, Ki-67 expression analyses using immunohistochemistry (IHC) are invasive and frequently influenced by tissue sampling quality. In this study, we assessed the feasibility of noninvasive magnetic resonance imaging (MRI) in predicting the Ki-67 labeling indices (LIs). A total of 51 lung cancer patients, including 42 non-small cell lung cancer (NSCLC) cases and nine small cell lung cancer (SCLC) cases, were enrolled in this study. Quantitative MRI parameters from conventional diffusion-weighted imaging (DWI), intravoxel incoherent motion (IVIM), and diffusion kurtosis imaging (DKI) were obtained, and their correlations with tumor tissue Ki-67 expression were analyzed. We found that the true diffusion coefficient (D value) from IVIM was negatively correlated with Ki-67 expression (Spearman r = -0.76, *P* < 0.001). The D values in the high Ki-67 group were significantly lower than those in the low Ki-67 group (0.90 ± 0.21 × 10^-3^ mm^2^/s vs. 1.22 ± 0.30 × 10^-3^ mm^2^/s). Among three MRI techniques used, D values from IVIM showed the best performance for distinguishing the high Ki-67 group from low Ki-67 group in receiver operating characteristic (ROC) analysis with an area under the ROC curve (AUROC) of 0.85 (95% CI: 0.73-0.97, *P* < 0.05). Moreover, D values performed well for differentiating SCLC from NSCLC with an AUROC of 0.82 (95% CI: 0.68-0.90), Youden index of 0.72, and F1 score of 0.81. In conclusion, D values were negatively correlated with Ki-67 expression in lung cancer tissues and can be used to distinguish high from low proliferation statuses, as well as SCLC from NSCLC.

## Introduction

Lung cancer was the most frequently diagnosed cancer and the leading cause of cancer death among males in 2012 1. Lung cancer survival rates are poor, with five-year survival rates of less than 20% 2. Ki-67 is a nuclear antigen present in most proliferating cells. Ki-67 is expressed during the active phases of the cell cycle, including the G1, G2, and S phases, and is a common marker used to detect tumor cell proliferation related to tumor invasiveness and prognoses. The Ki-67 labeling index (LI) has been widely used to predict the prognoses of breast cancer 3, glioma 4, and lung cancer 5. A meta-analysis demonstrated that high Ki-67 expression is associated with poor prognoses and disease progression in lung cancer patients. Ki-67 has been used as an independent biologic marker to predict lung cancer patient prognoses 6. However, the quantification of Ki-67 expression using immunohistochemistry (IHC) is invasive and frequently influenced by tissue sampling quality 7.

The application of magnetic resonance imaging (MRI) to lung cancer analyses is a relatively recent development yet is a rapidly growing field. Apparent diffusion coefficient (ADC) values derived from diffusion-weighted imaging (DWI) can reflect tumor cellularity. In recent years, ADC values were reported to be associated with Ki-67 expression in various tumors, such as breast cancer 8, 9, endometrial stromal sarcoma of the uterus 10, endometrial carcinoma 11, and lung cancer 12. However, ADC values are calculated using a mono-exponential model that is affected not only with the molecular movements of water but also by capillary microcirculation 13.

Intravoxel incoherent motion (IVIM) allows the separation of perfusion and molecular-based diffusion coefficients 14. By fitting multiple-b DWI data with a biexponential model, three parameters can be obtained from IVIM, including the true diffusion coefficient (D value), perfusion-related pseudodiffusion coefficient (D* value), and perfusion fraction (f value) 15-18. In addition, tumor tissue microenvironments are complicated, making the diffusion of water molecules behave in a non-Gaussian fashion, especially when b values are >1000 s/mm^2^ 19. A non-Gaussian diffusion model, diffusion kurtosis imaging (DKI), proposed by Jensen et al. 20, can measure tissue structures, including cellular compartments and membranes 18, 21-23.

To our knowledge, no studies have evaluated the associations between the quantitative parameters derived from IVIM and DKI and Ki-67 expression in lung cancer tissues. The difference in these IVIM and DKI parameters between small cell lung cancer (SCLC) and non-small cell lung cancer (NSCLC) are also largely unknown. Therefore, this study aimed to evaluate whether IVIM and DKI can predict Ki-67 expression levels in lung cancer tissues preoperatively and if IVIM and DKI quantitative values differ between SCLC and NSCLC.

## Materials and methods

### Patients

This prospective study was approved by the Clinical Research Ethics Committee of Huadong Hospital, Fudan University. All procedures performed in studies involving human participants were in accordance with the ethical standards of the institutional and/or national research committee and with the 1964 Helsinki Declaration and its later amendments or comparable ethical standards. Informed consent was obtained from all individual participants involved in the study.

From September 2016 to August 2018, patients who met the following criteria were included: (1) pulmonary nodule or mass diameters larger than 15 mm as detected by computed tomography (CT); (2) >50% of the tumors were solid; (3) Ki-67 tumor expression was available; (4) no previous treatments were given before MR examinations. The exclusion criteria were: (1) MRI contraindications present (n = 13); (2) unsatisfactory image qualities with server motion or distortion artifacts (n = 9); (3) A lack of pathologic results (n = 8). A total of 51 lung cancer patients were finally enrolled in this study, with 42 NSCLC and 9 SCLC patients. The characteristics of the enrolled patients are shown in **Table 1.**

### MRI acquisitions

All MRI examinations were performed with a 3-T MR scanner (MAGNETOM Prisma, Siemens Healthcare, Erlangen, Germany) using a 32-channel body coil and an integrated spine coil. After routine scanning that included coronal and transverse half acquisition single-shot turbo spin-echo (HASTE) T2-weighted imaging (T2WI), transversal turbo spin-echo (TSE) T2WI with fat suppression, and T1-volumetric interpolated breath-hold examination (VIBE), multi-b diffusion-weighted MRI scans (b = 0, 20, 60, 80, 150, 200, 400, 600, 800, 1200, 1600, 2000 s/mm^2^) with a single-shot echo-planar imaging pulse sequence in an axial orientation during free breathing was performed. The MRI protocols were listed in Table S1.

### Image analyses

All quantitative derived parameter maps were calculated using the prototype software Body Diffusion Toolbox (Siemens Healthcare, Erlangen, Germany). The DWI data were respectively post-processed with the mono-exponential and bi-exponential models. The ADC was calculated using the mono-exponential model from DWI with b values of 0 and 800 s/mm^2^, as show below:


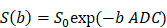


where S(b) represents the signal intensities at a specified b value, and S_0_ represents the signal intensities measured without radiofrequency saturations. Tumor regions of interest (ROIs) were drawn by outlining the tumor borders on ADC maps and showing the largest cross-section of the tumors. Necrotic areas and adjacent large vessels were avoided. Then the software-generated mean ADC values were recorded.

The IVIM parameters were calculated by fitting the acquired signal with 9 b-values (0, 20, 60, 80, 150, 200, 400, 600, and 800 s/mm^2^) into the IVIM model equation described by Le Bihan et al. 14:


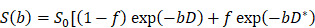


where D^*^ is the pseudo-diffusion coefficient representing the perfusion-related incoherent microcirculation, f is the pseudodiffusion fraction, and D is the true diffusion coefficient representing pure molecular diffusion.

DKI parameters including Dapp and Kapp, were obtained with six b-value signal intensities (b = 0, 600, 800, 1200, 1600, and 2000 s/mm^2^) fitted into the following equation 20:


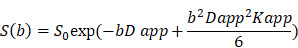


where Dapp represents diffusivity, and Kapp represents diffusion kurtosis.

Two radiologists (with 6 and 10 years of experience in MRI, respectively), who were blinded to the pathologic results, drew ROIs on the ADC maps and recorded the values of each parameter independently. The ROIs were automatically copied from the ADC maps to the corresponding IVIM-DKI parametric maps to obtain the values of D, D^*^, f, Dapp, and Kapp. Each lesion ROI was drawn twice and averaged to a mean value for analyses.

All DWI parameters were performed using a prototype Body Diffusion Toolbox (Siemens Healthcare, Erlangen, Germany). We selected 9 b-values (0, 20, 60, 80, 150, 200, 400, 600, and 800 s/mm^2^) to calculate DWI and IVIM parameters with the mono- and bi-exponential models, respectively. Six b-values (b = 0, 600, 800, 1200, 1600 and 2000 s/mm^2^) were selected for the DKI parameter calculations. The two blinded radiologists performed the following measurements. Tumor regions of interest (ROIs) were drawn by outlining tumor borders on ADC maps, showing the largest tumor cross-sections, and avoiding necrotic areas and adjacent large vessels by referring to T2WI and DWI images. The same ROIs were automatically copied to the D, D*, f, Dapp, and Kapp maps at the same level. All DWI parameters were measured synchronously.

### Immunohistochemistry

The immunohistochemical (IHC) analyses of Ki-67 expression were performed using the mouse monoclonal anti-human Ki-67 antibody (MIB-1, ZSGBBIO, Beijing, China). A pathologist with 14 years of experience in lung cancer pathology, blinded to the clinical and MRI data, assessed Ki-67 tumor expression. The percentage of Ki-67-positive cells was assessed by counting the number of stained nuclei per 100 tumor cells in the most representative areas (×400), corresponding to areas with the highest mitotic activity. Then, Ki-67 expression levels were divided into low (≤25%) and high (>25%) Ki67 expression groups, based on previous studies 24-26.

### Statistical analyses

Statistical analyses were conducted using SPSS 22.0 (IBM SPSS Statistics, USA) or GraphPad Prism 8.0 (Prism, USA) software. Quantitative data were expressed as the mean ± standard deviation (SD) or median and interquartile ranges based on the distribution. The interclass correlation coefficient (ICC) was used to evaluate reader reproducibility for parameter measurements (0.00-0.20, poor correlation; 0.21-0.40, fair correlation; 0.41-0.60, moderate correlation; 0.61-0.80, good correlation; and 0.81-1.00, excellent correlation). The correlations between the DWI-derived parameters and Ki-67 expression were analyzed using Spearman's rank correlations. The differences in DWI parameters between low and high Ki-67 expression and between SCLC and NSCLC were analyzed with a Student's t-test or Mann-Whitney U-test. Receiver operating characteristic (ROC) curve analyses were performed to determine the optimal cut-off values of these parameters for predicting high Ki-67 expression levels and lung cancer subtypes. The area under the ROC curve (AUCROC), sensitivity, specificity, Youden index, and F1 score were calculated. A *P* < 0.05 was considered statistically significant.

## Results

### Patients' characteristics

A total of 51 lung cancer patients were enrolled in this study, including 42 NSCLC and 9 SCLC patients. The median age was 64 (Range: 42-83). The mean Ki-67 values of all 51 patients was 39.7 ± 26.9% (range: 2%-90%). Thirty-one patients had Ki-67 expression values of > 25% (Table 1).

### DWI images and ADC, IVIM, and DKI maps of lung tumors in patients with NSCLC and SCLC

**Figure 1:**
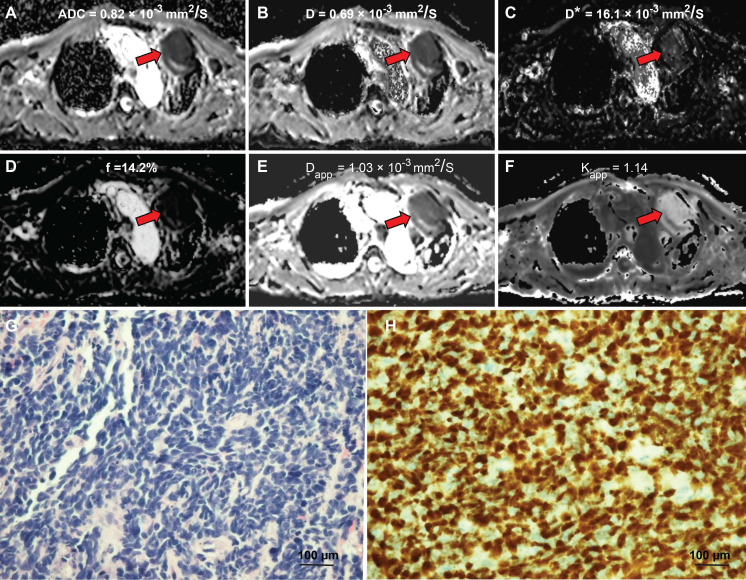
** A 58-year-old male diagnosed with small cell lung cancer.** (**A**) An axial ADC map showed a hypointense mass in the left pulmonary lobe (red arrow), with an ADC value of 0.82×10^-3^mm^2^/s. (**B**) A diffusion map (D) demonstrating a reduced D value (0.69×10^-3^ mm^2^/s). (**C**) A pseudodiffusion coefficient (D*) map demonstrating a D* value of 16.01x10^-3^ mm^2^/s. (**D**) A perfusion fraction (f) map showing an f value of 14.17%. (**E**) A diffusion map (Dapp) showing a Dapp value of 1.03×10^-3^ mm^2^/s. (**F**) A kurtosis map showing a Kapp value of 1.14. (**G**) Small cell lung cancer was confirmed by Hematoxylin and eosin (H&E) staining (magnification, × 400; scale bar, 100 µm). (**H**) Ki-67 immunohistochemical labeling shows that approximately 90% of cells are positive for nuclear staining (magnification, × 400, scale bar, 100 µm). ADC: Apparent diffusion coefficient; D: true diffusion coefficient, D* value: the perfusion-related pseudodiffusion coefficient; f: perfusion fraction; Kapp: diffusion kurtosis; Dapp: diffusivity.

**Figure 2:**
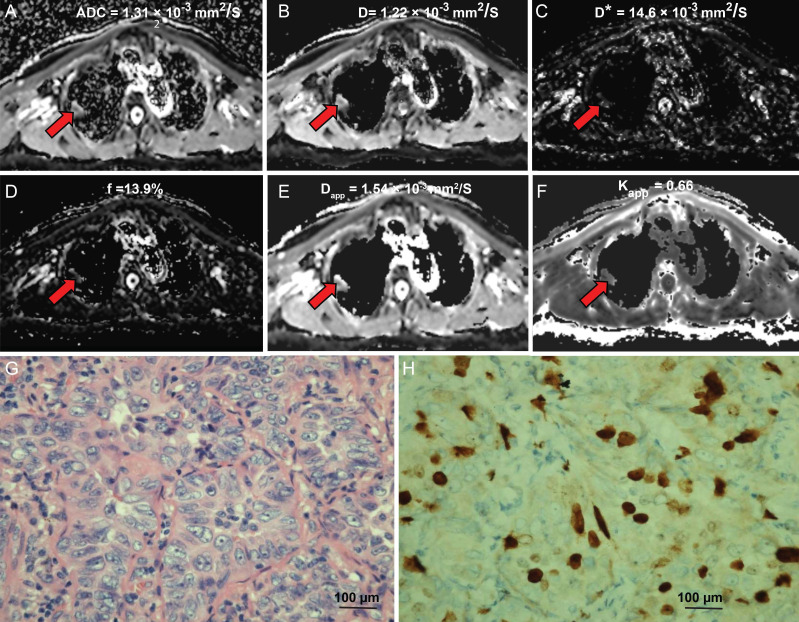
** An 80-year-old male diagnosed with lung adenocarcinoma.** (**A**) An axial ADC map shows a hypointense mass in the left pulmonary lobe (red arrow), with an ADC value of 1.13×10^-3^mm^2^/s. (**B**) A diffusion map demonstrating a D value of 1.22×10^-3^ mm^2^/s. (**C**) A pseudodiffusion coefficient map demonstrating a D* value of 14.63×10^-3^ mm^2^/s. (**D**) A perfusion fraction map showing an f value of 13.94%. (**E**) A diffusion map showing a Dapp value of 1.54×10^-3^ mm^2^/s. (**F**) A kurtosis map showing a Kapp value of 0.66. (**G**) Hematoxylin and eosin (H&E) staining confirms the mass to be lung adenocarcinoma (magnification, ×400, scale bar, 100 µm). (**H**) Ki-67 immunohistochemical labeling shows that approximately 10% of cells are positive for nuclear staining (magnification, × 400, scale bar, 100 µm).

The diffusion-weighted images of a typical NSCLC patient are shown in Figure S1. Tumor signals were attenuated with ascending b values. Figure 1 shows representative parametric maps of a 58-year-old male diagnosed with SCLC. The parametric maps (ADC, IVIM, and DKI) of an 80-year-old male diagnosed with lung adenocarcinoma are shown in Figure 2. Figures S2 and S3 show a 75-year-old male patient with lung squamous cell carcinoma and a 72-year-old male patient with large cell carcinoma, respectively. The parametric maps are followed by images of hematoxylin-eosin and Ki-67 immunohistochemical stained tissue sections.

### The associations between the quantitative IVIM and DKI values and Ki-67 expression

The interclass correlation coefficients (ICCs) for D* ranged from 0.33 - 0.84, and the ICCs of other parameters were 0.701-0.905. The ADC, D, and D* values of the high Ki-67 group were 1.11 ± 0.26 × 10^-3^ mm^2^/s, 0.90 ± 0.21 × 10^-3^ mm^2^/s, 16.66 ± 8.07 × 10^-3^ mm^2^/s, respectively, and were significantly lower than low Ki-67 group (ADC, D and D* values were 1.33 ± 0.30 × 10^-3^ mm^2^/s, 1.22 ± 0.30 × 10^-3^ mm^2^/s, 23.09 ± 12.70 × 10^-3^ mm^2^/s, respectively) (Table 2). However, the Kapp value of the high Ki-67 group (0.78 ± 1.98) was significantly higher than that of the low Ki-67 group (0.64 ± 0.15) (Table 2 and typical cases shown in Figures 1 and 2, and Figures S2 and S3). There were no significant differences in the f and Dapp values between the high and low Ki-67 groups.

**Figure 3:**
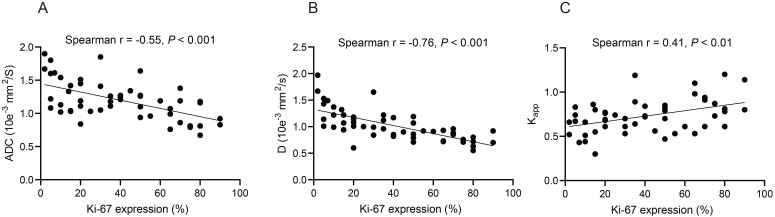
The significant correlations between the quantitative values of different magnetic resonance imaging (MRI) techniques and Ki-67 expression in lung tumor tissue sections. (**A**) Apparent diffusion coefficient (ADC); (**B**) True diffusion coefficient (D value); (**C**) Kapp values (diffusion kurtosis) from diffusion kurtosis imaging (DKI). Spearman's rank correlation was performed.

Spearman's rank correlation analyses revealed that the ADC and D values were negatively correlated with Ki-67 expression (r = -0.55 and -0.76, respectively, all *P* < 0.001) (Figures 3A and 3B). In contrast, Kapp values were positively correlated with Ki-67 expression (r = 0.41, *P* < 0.01) (Figure 3C). No significant correlations were found between D*, f, Dapp, and Ki-67 expression (Table S2).

**Figure 4:**
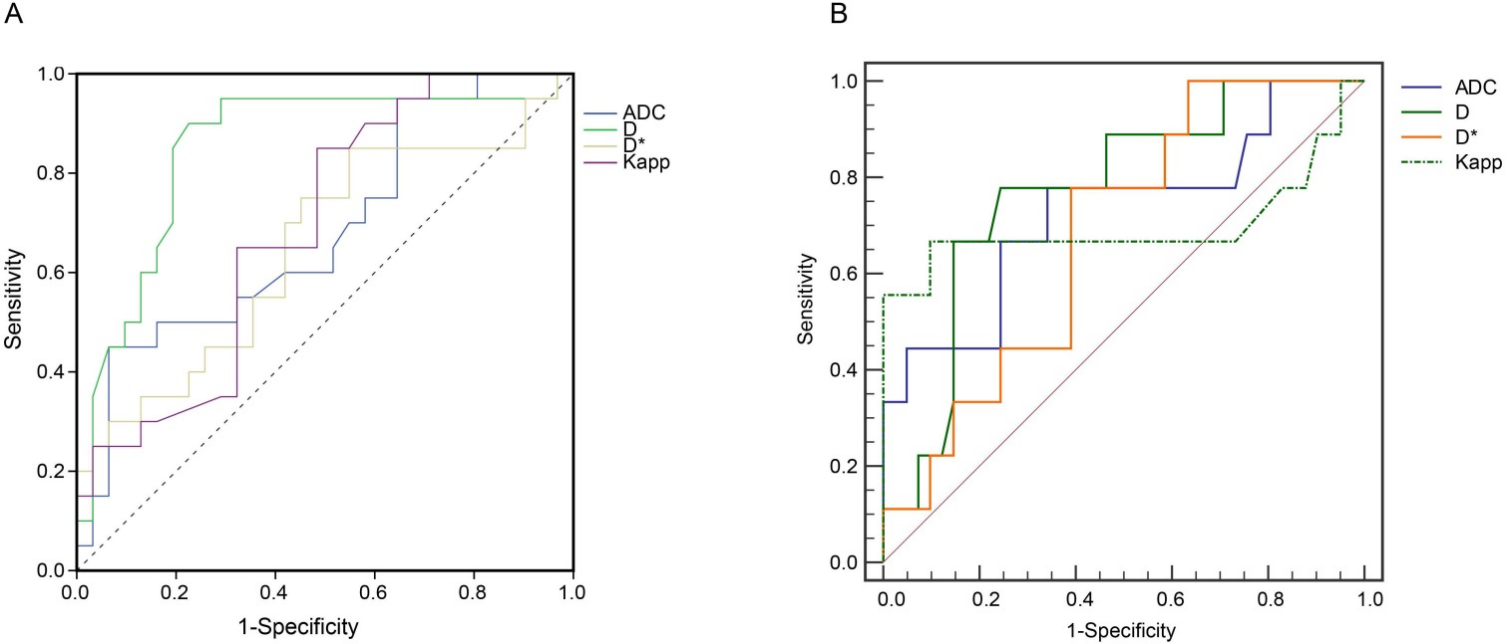
Receiver operating characteristic (ROC) curve analyses. (**A**) ROC curve analyses show the diagnostic performance of ADC, D, D*, Kapp in distinguishing the high Ki-67 group from the low Ki-67 group. D values produced the highest AUROC of 0.85 (95% CI: 0.73 - 0.97, *P* < 0.05). (**B**) ROC analysis of ADC, D, Dapp, and Kapp in differentiating SCLC and NSCLC. The 2 × 2 contingency analyses, including the cut-off values, sensitivities, specificities, Youden indices, and F1 scores, are listed in Table 3 and Table S4. The raw data are shown in Table S5. ADC: Apparent diffusion coefficient; D: true diffusion coefficient, D* value: perfusion-related pseudodiffusion coefficient; f: perfusion fraction; Kapp: diffusion kurtosis; Dapp: diffusivity; AUROC: Area under ROC curve; CI: confidence interval; SCLC: small cell lung cancer; NSCLC: non-small cell lung cancer.

The performance of ADC, D, D*, Dpp, and Kpp for distinguishing high Ki-67 group from the low Ki-67 group was then tested using AUROC curve analyses. Among the tested parameters, D values had the highest AUC of 0.85 (95% CI: 0.73-0.97, *P* < 0.001) in discriminating the high Ki-67 group from the low Ki-67 expression group (Figure 4A). In addition, D values yielded a sensitivity of 90.2%, a specificity of 77.4%, and a Youden index of 0.67 (Table 3).

### Quantitative MRI assessments for distinguishing SCLC and NSCLC

We found that the ADC, D, and Dapp values of SCLC were significantly lower than those of NSCLC (All *P* values <0.05) (Table S3). In contrast, the Kapp values of SCLC were significantly higher than those of NSCLC (0.90 ± 0.29 vs. 0.69 ± 0.14, *P* = 0.048). There were no significant differences in D* and f values between the two lung cancer types (all *P* > 0.05).

D values (Cutoff value = 0.85) performed well in discriminating SCLC and NSCLC with an AUC of 0.82 (95% CI: 0.68-0.92) (Figure 4B and Table S4). Compared with ADC, Dapp, and Kapp values, D values from IVIM yielded higher Youden index values (Table S4).

## Discussion

Differentiating SCLC from NSCLC (large cell carcinoma or basaloid squamous cell carcinoma) using noninvasive methods is essential since the therapeutic strategies and clinical prognoses are significantly different. Several noninvasive blood metabolomic and dynamic multiphase computed tomography (CT) methods have been recently developed to discriminate SCLC and NSCLC 27-30. However, blood metabolomic biomarker results that showed the differentiation of SCLC and NSCLC tumor types has not been confirmed with large cohort studies. Dynamic multiphase CT was shown to distinguish SCLC and NSCLC by evaluating tumor perfusion; however, significant radiation exposures restrict the use of this methodology. As a noninvasive, reusable technique, quantitative MRI is frequently used for tumor evaluations. In this study, we demonstrated for the first time that D values from IVIM had excellent accuracy in distinguishing NSCLC and SCLC.

Ki-67 is a common biomarker of tumor cell proliferation and has been shown to be associated with lung cancer prognoses and therapeutic efficacies 5, 31-33. In this study, we evaluated the associations between the DWI, IVIM, DKI parameters, and Ki-67 proliferation in tumor tissues of patients with lung cancer. We selected a cut-off of 25% for Ki-67 expression to differentiate the low and high Ki-67 groups 34. We found that D values from IVIM were negatively correlated with Ki-67 expression (Spearman's coefficient r = -0.76). D values between the high and low Ki-67 expression groups were significantly different. It is likely that the highly proliferative tumors (high Ki-67 expression) had higher inner structural complexities due to increased cellularity, vascular hyperplasia, and necrosis 23.

Although Dapp is corrected for non-Gaussian bias, it showed no significant correlation with Ki-67 expression. This might be due to tumor heterogeneity caused by vascular proliferation and internal necrosis. D* and f are IVIM perfusion-related parameters. No significant correlations were found between the D* or f values and Ki-67 expression in lung cancer, consistent with previous bladder cancer and sinonasal tumor studies 17, 18. These results indicated that extracellular components, such as average blood velocities, blood microcirculation, and capillary volumes, contributed little to lung tumor aggressiveness. In addition, we found poor D* and f value reproducibilities in lung cancer imaging, which might explain their low diagnostic performance in differentiating high and low Ki-67 groups.

D represents the true molecular diffusion of water without adsorption 14. In our study, D values performed well to identify lung tumors with high Ki-67 expression. Our results suggest that water molecule diffusion is affected more by tumor proliferation than by the complexity of the tumor microenvironment.

Previous studies 35, 36 have reported that ADC values were significantly different between SCLC and NSCLC. Our study found that ADC, D, and Dapp values of SCLC were dramatically lower than those of NSCLC. The potential histopathologic rationale might be that SCLCs are highly cellular, and the cells have large nuclei with scant cytoplasm; all of these factors could restrict diffusion motion reducing ADC, D, and Dapp values 37. As mentioned above, D* and f values were poorly repeatable. D* values were also related to blood flow velocities. The high variability of D* values could result from the dramatic differences in tumor vascularities among different tumor types 17. The f values are affected by the T2 contributions in both the perfusion and pure molecular diffusion compartments. Therefore, measuring IVIM D values could be used as a noninvasive approach to distinguish SCLC and NSCLC.

Our study had several limitations. First, the distribution of lesion types was uneven, and the number of patients with SCLC was relatively small compared with the number of patients with NSCLC. When the dataset was unbalanced, traditional classification metrics, such as AUROCs and sensitivities, might be overfitted. The F1 score is the weighted average of precision and recall; data distribution is considered and particularly suitable for unbalanced datasets 38. Although the F1 score is acceptable for our analyses, further validations with other independent cohorts are still required. Second, ROIs drawn on the parametric maps might not correspond well with the Ki-67 expression in histologic specimens. MR-guided biopsies could solve this problem. Third, the optimal b-value combination for lung IVIM and DKI analyses was unclear. Adding more b values could improve fitting accuracies, improving IVIM or DKI parameter performance predictions. However, acquisition times would be increased accordingly.

## Conclusions

In summary, ADC, D, and Kapp values were significantly associated with Ki-67 proliferation statuses in patients with lung cancer. D values obtained from IVIM had the highest diagnostic performance in distinguishing high and low Ki-67 statuses. Moreover, D values to differentiate SCLC and NSCLC performed well. Our study provided a noninvasive approach to predict Ki-67 expression and distinguish different lung cancer types.

## Supplementary Material

Supplementary figures and tables.

## Figures and Tables

**Table 1:** Characteristics of patients in this study

Characteristics	Value	Percentage	*P* value
**Sex**			
Male	27	52.9%	0.69
Female	24	47.1%	
**Age**			
Median (range)	64 (42 - 83)		
Ki67 (Mean ± SD)	39.7 ± 26.9		
>25% (n)	31	60.8%	0.05
≤25% (n)	20	39.2%	
**Pathological feature**			
***NSCLC (Non-small cell lung cancer)***	42	82.4%	
Adenocarcinoma	28		
Squamous cell carcinoma	12		< 0.001
Large cell carcinoma	2		
***SCLC (Small cell lung cancer)***	9	17.7%	

Data are number and percentage or mean and standard deviation (SD). A two-sample proportion Z-Test was conducted to compare the differences between categorical variables.

**Table 2:** Comparison of ADC, D, D*, f, Kapp, and Dapp values of lung tumors with low and high Ki-67

Parameters	Low Ki-67 (n = 21)	High Ki-67 (n = 30)	* P*
ADC (×10^-3^ mm^2^/s)	1.33 ± 0.30	1.11 ± 0.26	0.012
D (×10^-3^ mm^2^/s)	1.22 ± 0.30	0.90 ± 0.21	0.000
D^*^ (× 10^-3^ mm^2^/s)	23.1 ± 12.7	16.7 ± 8.07	0.031
f (%)	30.3 ± 15.2	26.9 ± 16.5	0.48
Kapp	0.64 ± 0.15	0.78 ± 1.98	0.011
Dapp (× 10^-3^ mm/s)	1.91 ± 0.51	1.74 ± 0.68	0.34

Data are mean ± standard deviation (SD). ADC: apparent diffusion coefficient; D: true diffusion coefficient, D* value: the perfusion-related pseudodiffusion coefficient; f: perfusion fraction; Kapp: diffusion kurtosis; Dapp: diffusivity.

**Table 3:** ROC curve analysis for ADC, D, D^*^, and Kapp values in discriminating lung cancers with high and low Ki-67 expression

Parameters	Cut-off value	Sensitivity (%)	Specificity (%)	AUROC (95% CI)	Youden index	*P*
ADC (×10^-3^mm^2^/s)	1.42	45.1	93.5	0.68 (0.53 -0.84)	0.39	0.029
D (×10^-3^mm^2^/s)	0.98	90.2	77.4	0.85 (0.73 - 0.97)	0.67	0.000
D*(×10^-3^mm^2^/s)	13.1	85.2	45.2	0.65 (0.49 -0.81	0.31	0.073
Kapp	0.78	51.6	85	0.69 (0.55 - 0.83)	0.37	0.023

ROC: receiver operating characteristic; ADC: apparent diffusion coefficient; D: true diffusion coefficient, D* value: the perfusion-related pseudodiffusion coefficient; f: perfusion fraction; Kapp: diffusion kurtosis; Dapp: diffusivity; AUROC: Area under ROC curve; CI: confidence interval.
