# Supplementary material for: A Noninvasive Assessment of Tumor Proliferation in Lung cancer Patients using Intravoxel Incoherent Motion Magnetic Resonance Imaging
Source: J Cancer. 2021 Jan 1;12(1):190–7. doi: 10.7150/jca.48589 (PMC7738818; doi:10.7150/jca.48589)
Supplement: Supplementary file 1 — Supplementary figures and tables. [file jcav12p0190s1.pdf]

Supplemental Materials

The Noninvasive Assessment of Ki-67 Expression in Lung cancer Patients Through Intravoxel Incoherent Motion Magnetic Resonance Imaging

Yu Zheng[1#], Wenjun Huang[1#], Xuelin Zhang[2], Chen Lu[3], Caixia Fu[4], Shihong Li[1]*, Guangwu Lin[1]*

[1]Department of Radiology, Huadong Hospital Affiliated to Fudan University, Shanghai, 200040, China

[2]Department of Thoracic Surgery, Huadong Hospital Affiliated to Fudan University, Shanghai, 200040, China

[3]Department of Pathology, Huadong Hospital Affiliated to Fudan University, Shanghai, 200040, China

[4]Siemens Shenzhen Magnetic Resonance Ltd., Shenzhen, Guangdong Province, 518057, China

Supplemental figures

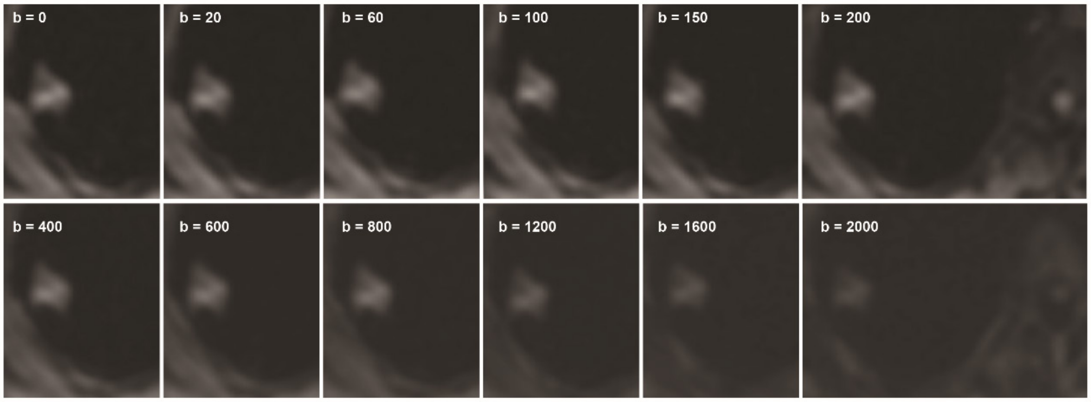

Figure S1. The diffusion-weighted images with multiple ascending b values for a case of non-small cell lung cancer (NSCLC). The signal intensity of the nodule was gradually attenuated.

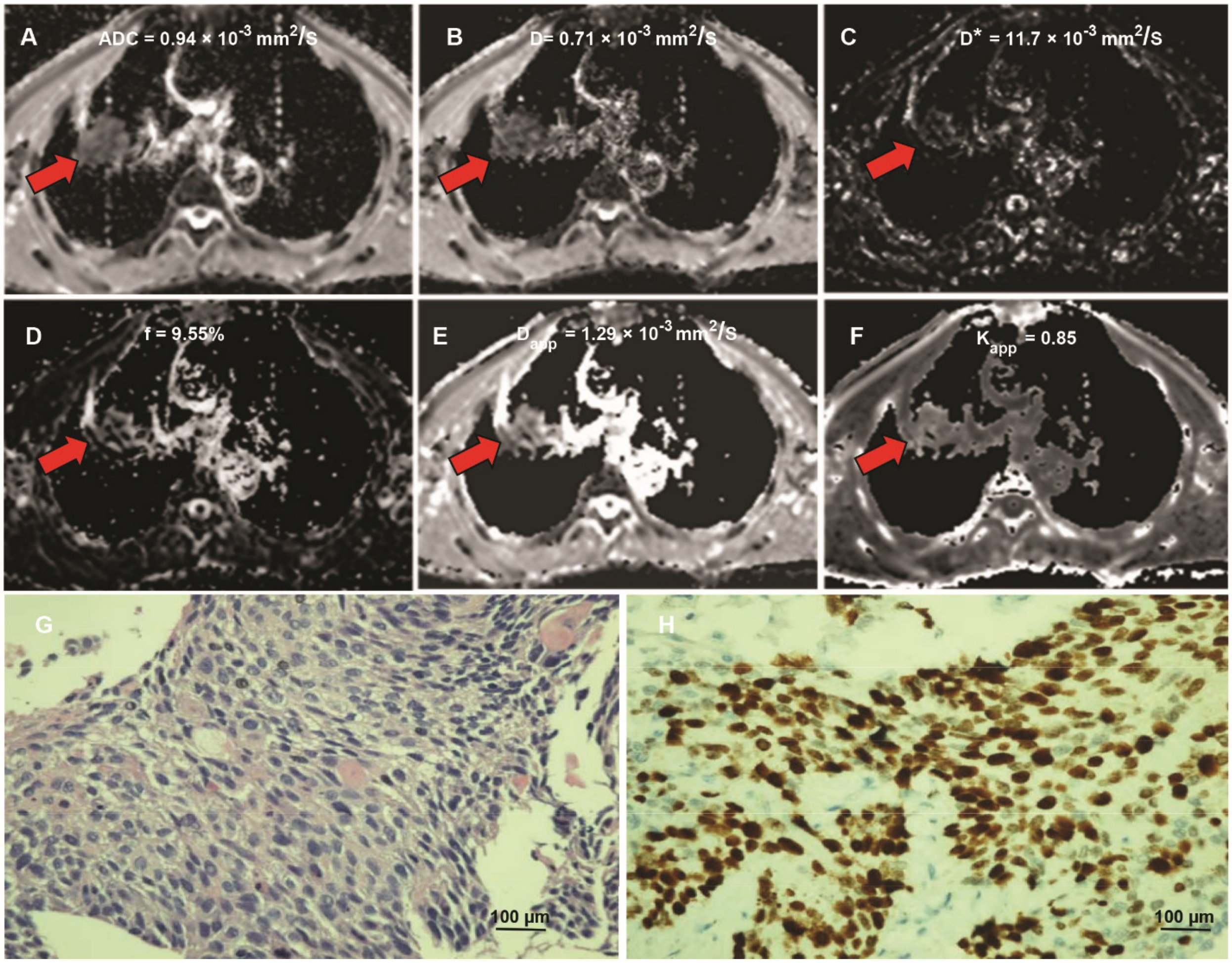

Figure S2. A 75-year-old male patient with lung squamous cell carcinoma. (**A**) An axial ADC map showed that a hyporintense mass is located in the right pulmonary upper lobe (red arrow), with an ADC value of 0.93 × 10^{-3} mm^2/s. (**B**) diffusion map demonstrating reduced D value (0.72 × 10^{-3} mm^2/s). (**C**) pseudodiffusion coefficient (D*) map demonstrating D* value (11.73 × 10^{-3} mm^2/s). (**D**). perfusion fraction (f) map showing f value of 9.56%. (**E**) diffusion map (Dapp) showing Dapp value of 1.28 × 10^{-3} mm^2/s), and (**F**) kurtosis map showing the Kapp value of 0.85. (**G**) Haematoxylin-eosin staining confirms the mass as a squamous cell carcinoma (magnification, × 400, 100 µm). (**H**) Ki-67 immunohistochemical labelling shows that approximately 80% of cells are positive for nuclear staining (magnification, × 400, 100 µm).

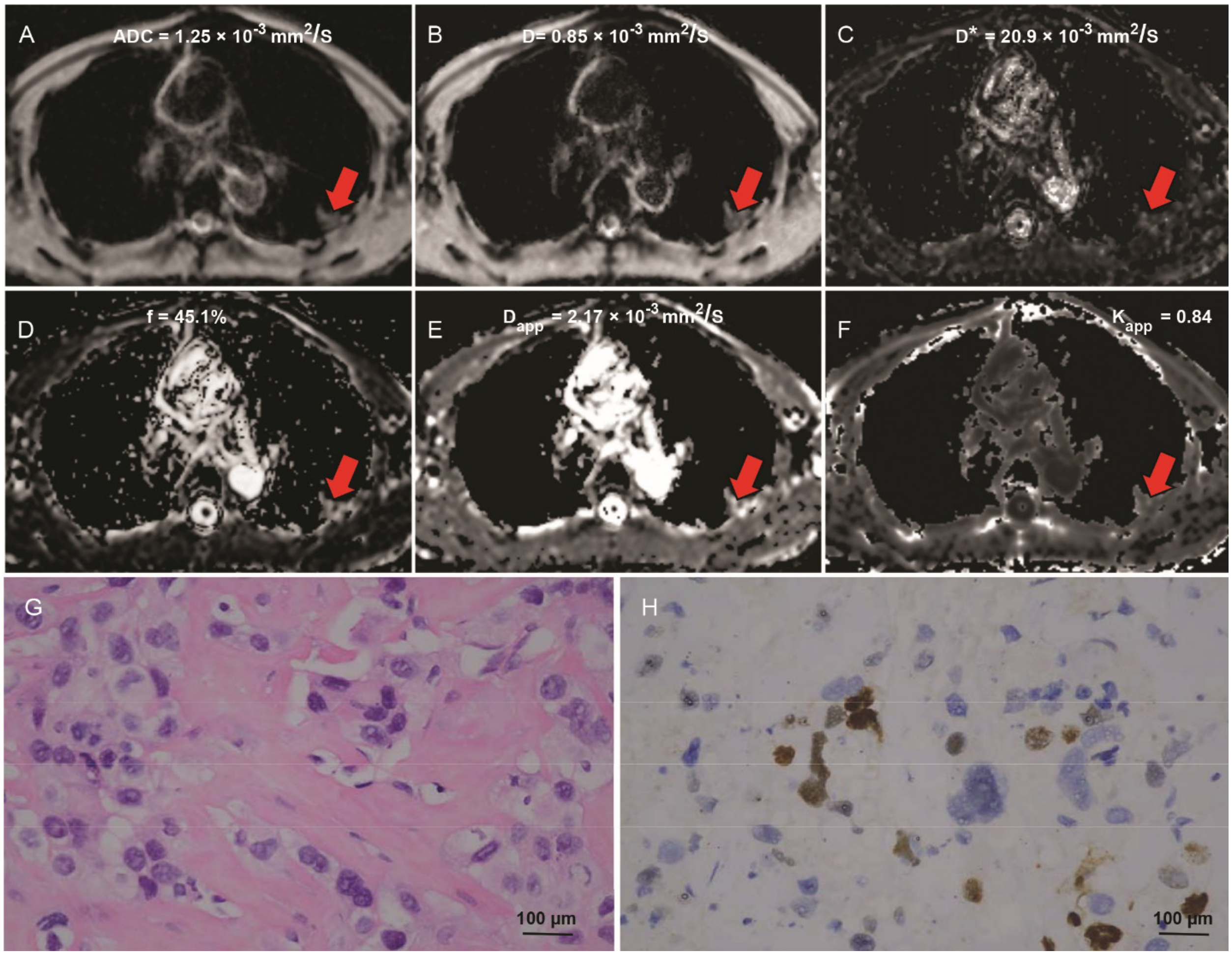

Figure S3. A 72-year-old male patient with large cell carcinoma. (**A**) An axial ADC map showed a hyporintense pulmonary nodule is located in the left pulmonary upper lobe (red arrow), with an ADC value of 1.25 ×10^{-3} mm^2/s. (**B**) diffusion map (D) demonstrating reduced D value (0.85 × 10^{-3} mm^2/s). (**C**) pseudodiffusion coefficient (D*) map demonstrating D* value (20.90 ×10^{-3} mm^2/s). (**D**). perfusion fraction (f) map showing the f value of 45.1%. (**E**)diffusion map (Dapp) showing the Dapp value (2.17×10^{-3} mm^2/s), and (**F**) kurtosis map showing the Kapp value of 0.84. (**G**) Haematoxylin-eosin staining confirms the nodule as a large cell carcinoma (magnification, × 400, 100 µm). (**H**) Ki-67 immunohistochemical labelling shows that approximately 40% of cells are positive for nuclear staining (magnification, × 400, 100 µm)

Table S1 MRI sequence parameters

Sequence	T2WI-Haste	T2WI-FS -triggered	T1-VIBE	Conventional DWI	IVIM-DKI
Plane	transverse	transverse	transverse	transverse	transverse
TR/TE, ms	1400/90	2500/80	3.97/1.23	3000/41	4000/54
Slice thickness (mm)	5	5	3	5	5
Flip Angle	160	101	9		
Intersection gap	30%	20%	20%	20%	20%
FOV (mm)	400	400	440	380	380
Matrix	320 × 320	384 × 384	320 × 195	134 × 100	128 × 128
TA (second)	40	155	15	127	504

Abbreviations: MRI: Magnetic resonance imaging; T2WI: T2 weighted image; T2WI-FS: T2 weighted image-fat-suppressed; T1-VIBE: T1-volumetric interpolated breath-hold examination; DWI: diffusion weighted imaging; IVIM-DKI: intravoxel incoherent motion-diffusion kurtosis imaging; TR/TE: repetition time/echo time; FOV: Field-of-view; TA:

Table S2 The correlations between MRI parameters and Ki-67 LI

Parameters	Coefficient r	*P*
ADC ($\times 10^{-3}$ mm^2/s)	-0.55	0.000
D ($\times 10^{-3}$ mm^2/s)	-0.76	0.000
D^* ($\times 10^{-3}$ mm^2/s)	-0.26	0.069
f (%)	-0.22	0.121
Kapp	0.41	0.001
Dapp ($\times 10^{-3}$ mm^2/s)	-0.22	0.116

Note: Spearman's correlation was performed. MRI: Magnetic resonance imaging; ADC: apparent diffusion coefficient; D: true diffusion coefficient, D* value: the perfusion-related pseudodiffusion coefficient; f: perfusion fraction; Kapp: diffusion kurtosis; Dapp: diffusivity.

Table S3. Comparison of MRI values between NSCLC and SCLC

Parameters	NSCLC (n = 42)	SCLC (n = 9)	*P*
ADC ($\times 10^{-3}$ mm^2/s)	1.24 ± 0.28	1.00 ± 0.25	0.025
D ($\times 10^{-3}$ mm^2/s)	1.07 ± 0.29	0.79 ± 0.16	0.003
D^*($\times 10^{-3}$ mm^2/s)	20.3 ± 10.9	13.8 ± 5.73	0.103
f (%)	30.2 ± 16.1	19.4 ± 12.1	0.079
Kapp	0.69 ± 0.14	0.90 ± 0.29	0.048
Dapp($\times 10^{-3}$ mm/s)	1.91 ± 0.59	1.32 ± 0.51	0.005

MRI: Magnetic resonance imaging; NSCLC: Non-small cell lung cancer; SCLC: small cell lung cancer; ADC: apparent diffusion coefficient; D: true diffusion coefficient, D* value: the perfusion-related pseudodiffusion coefficient; f: perfusion fraction; Kapp: diffusion kurtosis; Dapp: diffusivity;

Table S4 ROC analysis for ADC, D, Dapp and Kapp values in differentiating SCLC from NSCLC

Parameters	Cut-off value	Sensitivity (%)	Specificity (%)	AUC (95% CI)	Youden index	F1 score	*P*
ADC ($\times10^{-3}mm^2/s$)	0.84	95.2	44.4	0.73 (0.59 - 0.85)	0.39	0.69	0.025
D ($\times10^{-3}mm^2/s$)	0.85	83.3	77.8	0.82 (0.68 - 0.90)	0.72	0.81	0.003
Dapp ($\times10^{-3}mm^2/s$)	1.45	76.2	77.8	0.68 (0.53 - 0.81)	0.54	0.65	0.005
Kapp	0.87	66.7	92.9	0.71 (0.55 - 0.82)	0.60	0.68	0.048

ROC: receiver operating characteristic; SCLC: small cell lung cancer; NSCLC: non-small cell lung cancer; ADC: apparent diffusion coefficient; D: true diffusion coefficient, D* value: the perfusion-related pseudodiffusion coefficient; f: perfusion fraction; Kapp: diffusion kurtosis; Dapp: diffusivity; AUROC: Area under ROC curve; CI: confidence interval.

Table S5 Raw data for receiver operating characteristic (ROC) curve analysis

Diagnosis	ADC	D	D2	Kapp
0	1.16	0.88	18.75	0.61
0	1.85	1.65	24.73	0.7
0	1.21	0.82	11.8	0.72
0	1.02	0.99	18.12	0.51
0	1.08	1.01	21.78	0.83
0	1.27	1.17	11.44	0.73
0	1.13	1.22	14.63	0.66
0	1.14	0.94	32.97	0.8
0	1.19	0.96	18.1	0.94
0	1.26	1.22	10.24	0.89
0	1.61	1.49	19.8	0.43
0	1.62	1.43	21.41	0.74
0	1.25	0.85	20.9	0.84
0	1.19	0.91	27.83	0.61
0	1.9	1.97	58.05	0.52
0	1.45	1.01	7.32	0.69
0	0.96	0.92	22.2	0.53
0	0.84	0.6	35.23	0.61
0	0.93	0.92	31.13	0.8
0	1.18	0.63	12.31	0.61
0	0.94	0.71	11.73	0.85
0	1.1	1.07	19.96	0.79
0	1.22	1.14	13.79	0.67
0	1.19	1.14	38.14	0.76
0	0.81	0.72	10.8	0.78
0	1.26	1.19	19.15	0.82
0	0.86	0.85	5.45	0.91
0	0.93	0.84	12.3	0.85
0	1.11	0.96	33.79	0.64
0	1.34	0.9	32.54	0.56
0	1.03	1	31.9	0.74
0	1.64	0.86	7.61	0.7
0	1.51	1.18	26.47	0.77
0	1.16	0.8	7.31	0.81
0	1.03	1.07	18.7	0.55
0	0.81	0.71	25.26	0.73
0	0.96	0.91	17.4	0.79
0	1.39	1.11	15.4	0.8
0	0.9	0.67	12.39	0.89
0	1.01	0.81	9.23	0.64
0	1.42	1.22	36.44	0.3
1	0.67	0.55	12.21	1.2

1	0.98	0.84	20.62	0.61
1	1.41	1.12	10.36	1.19
1	0.83	0.7	16.03	1.14
1	0.76	0.73	16.11	0.98
1	0.99	0.93	15.48	1.1
1	1.27	0.79	8.18	0.47
1	1.07	0.72	3.99	0.53
1	0.79	0.76	21.62	0.87

Note: 0: for non-small cell lung cancer (NSCLC), and 1: for small cell lung cancer (SCLC)
